# Optimum management of asymptomatic moderate-severe degenerative mitral regurgitation: a role for T1 mapping in risk stratification?

**DOI:** 10.1186/1532-429X-16-S1-P238

**Published:** 2014-01-16

**Authors:** Nicola C Edwards, Mengshi Yuan, Isabelle K Good, William E Moody, Richard P Steeds

**Affiliations:** 1 Cardiovascular Medicine, University of Birmingham, Birmingham, UK; 2 Cardiology, Queen Elizabeth Hospital, Birmingham, UK

## Background

The optimum timing of surgery in asymptomatic patients with severe degenerative mitral regurgitation (MR) is controversial and there are no randomised data to separate early repair from watchful waiting. Chronic volume overload may be a stimulus for progressive myocardial fibrosis, which could potentially be an additional marker for left ventricular (LV) recovery after surgery.

## Methods

In total, 24 patients (mean age 62 +/- 16 years) with degenerative moderate or severe asymptomatic MR with no class 1 indication for surgery were recruited from a dedicated valve clinic in a University Hospital and compared with gender matched controls. All subjects underwent cardiac MRI (1.5T Siemens Avanto) for assessment of LV volumes, deformation (SPAMM tagging), late gadolinium enhancement (LE) and T1-mapping using a modified look-locker inversion recovery sequence (MOLLI) before and 15 minutes post gadolinium (0.1 mmol/Kg) for myocardial extracellular volume (ECV). Global ECV was the average measure from basal and mid short axis slices excluding LGE indicative of infarcted myocardium.

## Results

**Figure 1:**
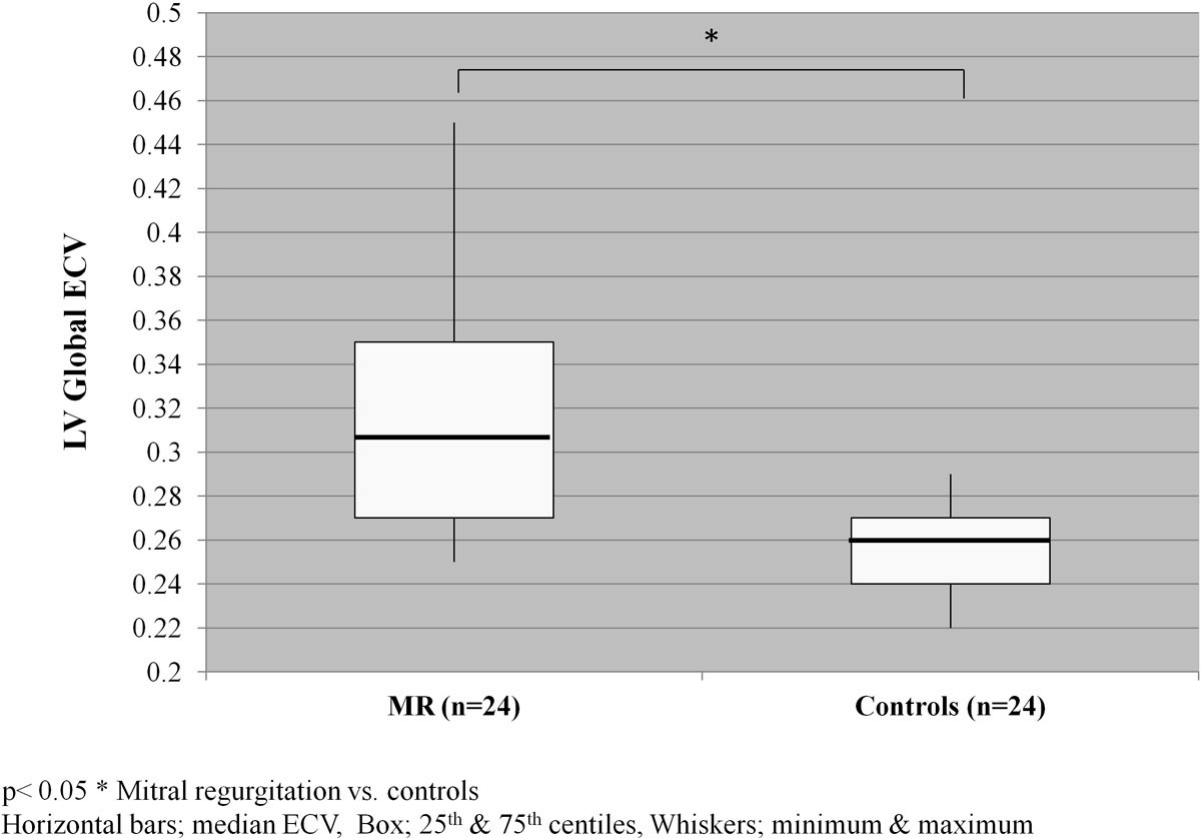
**Global extraceullar volime in patients with moderate-severe degenerative mitral regurgitation and controls**.

Patients with MR (mean regurgitant volume 42 ml ± 20) had impaired longitudinal and circumferential systolic deformation (Table 1) and increased global ECV (Figure 1). These changes preceded a difference in LV ejection fraction, MAPSE (Table 1) or increase in NT-proBNP (median 85 ng/L IQR 280). Global ECV was inversely correlated with Vo2 max (r = - 0.771, p < 0.05). Nine patients had patchy diffuse LE in non-coronary artery territories primarily within the basal segments with an increased ECV compared to remote "normal" myocardium.

**Table 1:** CMR parameters of structure and function

	MR	Healthy controls
LVEDV (ml/m2)	89 (18)	65 (12)*

LVESV (ml/m2)	28 (7)	20 (6)

LV mass index (g/m2)	68 (13)	57 (13)

LVEF (%)	70 (9)	72 (6)

MAPSE (mm)	14 (5)	16 (3)

Peak longitudinal systolic strain (%)	12.0 (3.3)	18.5 (5.0)*

Peak longitudinal systolic strain rate (s-1)	0.60 (0.14)	1.04 (0.42)*

Peak basal circumferential strain (%)	10.9 (3.3)	15.5 (3.1)*

Peak basal circumferential strain rate (s-1)	0.63 (0.2)	0.8 (0.1)*

Peak mid circumferential strain (%)	15.2 (5.0)	18.1 (2.4)

Peak mid circumferential strain rate (s-1)	0.8 (0.2)	1.0 (0.2)*

Data = mean (SD), *p < 0.05

## Conclusions

Patients with MR demonstrate a spectrum of myocardial fibrosis associated with reduced longitudinal and circumferential deformation and reduced peak VO2 max. These changes occur before changes in conventional markers of LV structure and function or an increase in BNP. The routine use of CMR may provide a rationale for improved risk stratification in patients with asymptomatic moderate-severe primary MR to optimise the risk/benefit of early surgery.

## Funding

Nil.

